# Updates on the Biofunctionalization of Gold Nanoparticles for the Rapid and Sensitive Multiplatform Diagnosis of SARS-CoV-2 Virus and Its Proteins: From Computational Models to Validation in Human Samples

**DOI:** 10.3390/ijms24119249

**Published:** 2023-05-25

**Authors:** Rodica Elena Ionescu

**Affiliations:** Light, Nanomaterials and Nanotechnology (L2n) Laboratory, CNRS EMR 7004, University of Technology of Troyes, 12 Rue Marie Curie, CS 42060, CEDEX, 10004 Troyes, France; elena_rodica.ionescu@utt.fr; Tel.: +33-3-2575-9728; Fax: +33-3-2571-8456

**Keywords:** SARS-CoV-2 virus, biofunctionalized gold nanoparticles, multidetection platforms, computational prediction, dry and wet chemistry, lateral flow immunoassays, commercial rapid tests

## Abstract

Since the outbreak of the pandemic respiratory virus SARS-CoV-2 (COVID-19), academic communities and governments/private companies have used several detection techniques based on gold nanoparticles (AuNPs). In this emergency context, colloidal AuNPs are highly valuable easy-to-synthesize biocompatible materials that can be used for different functionalization strategies and rapid viral immunodiagnosis. In this review, the latest multidisciplinary developments in the bioconjugation of AuNPs for the detection of SARS-CoV-2 virus and its proteins in (spiked) real samples are discussed for the first time, with reference to the optimal parameters provided by three approaches: one theoretical, via computational prediction, and two experimental, using dry and wet chemistry based on single/multistep protocols. Overall, to achieve high specificity and low detection limits for the target viral biomolecules, optimal running buffers for bioreagent dilutions and nanostructure washes should be validated before conducting optical, electrochemical, and acoustic biosensing investigations. Indeed, there is plenty of room for improvement in using gold nanomaterials as stable platforms for ultrasensitive and simultaneous “in vitro” detection by the untrained public of the whole SARS-CoV-2 virus, its proteins, and specific developed IgA/IgM/IgG antibodies (Ab) in bodily fluids. Hence, the lateral flow assay (LFA) approach is a quick and judicious solution to combating the pandemic. In this context, the author classifies LFAs according to four generations to guide readers in the future development of multifunctional biosensing platforms. Undoubtedly, the LFA kit market will continue to improve, adapting researchers’ multidetection platforms for smartphones with easy-to-analyze results, and establishing user-friendly tools for more effective preventive and medical treatments.

## 1. Introduction

The contagious respiratory SARS-CoV-2 RNA virus emerged in early 2020 and continues to cause moderate to severe infections in many people [1], where the neutrophil-to-lymphocyte ratio and elevated serum creatinine biomarkers were linked to advanced disease symptoms and mortality [2]. The virus has structural proteins (spike (S), nucleocapsid (N), membrane (M), and envelope (E)) and non-structural proteins (RNA polymerase, 3CL protease) that serve as sensitive biomarkers, which are expressed during the viral infection [3]. These proteins are detected using either time-consuming conventional molecular techniques (e.g., an enzyme-linked immunosorbent assay (ELISA) [4,5], polymerase chain reaction (PCR) [6]) and/or rapid immunochromatographic tests (less than 20 min) known as lateral flow assays, which are easy to handle both for physicians and for the untrained public. To evaluate the presence/absence of a viral infection, different bodily fluids (whole blood, serum, saliva, nasal secretions, urine) are collected and analyzed. Unfortunately, commercial LFAs have lower sensitivity than PCRs and false negative results are often obtained at low viral loads. Therefore, improved detection techniques that are low-cost, selective, and provide reproducible results are urgently needed [7,8,9].

In this context, nanotechnology [10,11] is in high demanded for the rapid detection and treatment of SARS-CoV-2 infection [12]. Among the different nanomaterials [13], inorganic metal nanoparticles, especially noble metals, have made a significant contribution to controlling the spread of COVID-19 and assisting detection, immunodiagnosis, drug development, and photocatalytic viral inactivation. More specifically, colloidal gold nanoparticles (AuNPs) are intensively used in “in vitro” biomedical applications due to their unique physical and optical properties including localized surface plasmon resonance (LSPR) in the visible spectrum, fluorescence resonance energy transfer (FRET), and surface-enhanced Raman scattering (SERS) [14]. Moreover, AuNPs are among the most stable metal nanoparticles; they are highly biocompatible, easy to synthesize, and have numerous biomolecule functionalization abilities [15,16]. They have contributed significantly to the development of varied biosensors with highly improved analytical performance (low detection limits, sensitivity, response times), vaccine formulation [17,18,19,20,21,22], and the large-scale manufacturing of rapid tests [23,24,25]. 

In the presence of biomolecules, the optical properties of AuNPs change according to their size and shape, leading to different absorption bands that confirm the immobilization strategy step by step [26,27]. To date, the conjugation of AuNPs with viral proteins has typically been carried out by the physical electrostatic interaction at the pH value, varied within a range of ±0.5 around the protein’s isoelectric point [28]. Conversely, the other pH values exhibit a purple or white-gray color, indicating their aggregation and destructive effect on gold particles.

Nowadays, AuNPs are used for the ultrasensitive detection of varied targets [29] such as enzymes [30,31], proteins [32], DNA [33,34], cells [35], viruses [36,37,38,39,40,41], and bacteria [42,43]. Moreover, promising therapeutic approaches have just begun using AuNPs as antiviral agents capable of trapping viruses [44,45,46,47], in anticoagulant therapy [48], in photothermal therapy for breast cancer [49], and in the treatment of inflammatory bowel disease [50]. Preliminary studies using AuNPs as an anticancer agents [51] and medication [52] have also been reported, since bioinspired AuNPs can reach tumors via their enhanced permeability and retention effect [53].

AuNPs have also been used as signal transducers, acting as remarkable optical signal amplifiers, current amplifiers and resonance light-scattering supports in the fabrication of ultrasensitive biosensors, significantly improving the sensitivity of diagnoses. There are examples based on various multilevel optical (reflectometry [54], surface-enhanced Raman scattering (SERS) [55,56], localized surface plasmon resonance (LSPR) [57,58]) electrochemical (non-faradaic electrochemical impedance spectroscopy (n-EIS) [59,60], faradaic EIS [61], square-wave voltammetry (SWV) [62]) and piezoelectric [63]/acoustic [64] sensing platforms for the SARS-CoV-2 virus [65]. Interestingly, the rapid “in-tube” naked-eye detection of SARS-CoV-2 RNA was locally recorded in the presence of AuNPs capped with thiol-modified antisense oligonucleotides specific for the N gene and monitored by UV–visible absorbance spectroscopy, transmission electron microscopy, and hyperspectral microscopy [66]. Recently, the implementation of optical nanosensors based on AuNPs functionalized with oligonucleotides was proposed for the rapid and ultrasensitive detection of SARS-CoV-2 traces (1 ng/mL) on food surfaces and for viral prevention [67]. Additionally, the first attempts to use electrochemical sensing platforms for the internet of healthcare things (IoHT) have been made via the implementation of wearable biosensor patches with internet connection; these enable the rapid recording of viral symptoms and further data transmission to medical services in real time, facilitating the fastest drug treatments of positive individuals [68]. 

In the context of the COVID-19 pandemic, portable lateral flow assays based on paper strips are in high demand for the naked-eye viral detection obtained over different interconnected membranes (the sample pad, the conjugate pad, the nitrocellulose (NC) membrane, and the absorbent pad) assembled in a sandwich form, when the tested sample moves on the strip via capillary action [69]. LFAs have been intensively optimized in terms of different parameters: the sample drop volume, type of sample pad/conjugation pad/NC flow membrane pore size, blocking conditions, antibody coating concentration, incubation time, running buffers/effects of salts, storage temperature/protective packaging bags, and signal stability. They have been used for the detection of SARS-CoV-2 Ab in whole blood, serum, and plasma. To confirm a SARS-CoV-2 infection, colloidal AuNP-conjugated viral Ab [70] were used for LFA tests. IgA/IgG/IgM Ab [71] were typically used to draw the test line (T-line) and the control line (C-line) to validate the results [72] with the naked eye and with much more sensitive smartphone read-out facilities and high-sensitivity synchrotron X-ray fluorescence imaging [73]. When the sample pad was modified with SARS-CoV-2 receptor binding domain (RBD) Ag from an infected individual, it interacts with the specific AuNP-Ab probe dropped on the conjugate pad, resulting in the formation of immunocomplexes that migrate into the zone of detection, along the NC membrane, with the red T-line becoming visible within about 20 min. 

More advanced LFA configurations propose either the use of glycan-based LFA diagnostics, as it can detect both the active and deactivated SARS-CoV-2 virus, with results similar to those obtained with other tests, or the integration of isothermal nucleic acid amplification with LFA, which highlights the possibility of applying genetic testing to affordable point-of-care systems. These LFAs may overcome the current limitations of expensive conventional methods, namely PCR and its related quantitative polymerase chain reaction (qPCR) [74], and the latest generation of digital PCR (dPCR) tests [75]. 

**Figure 1:**
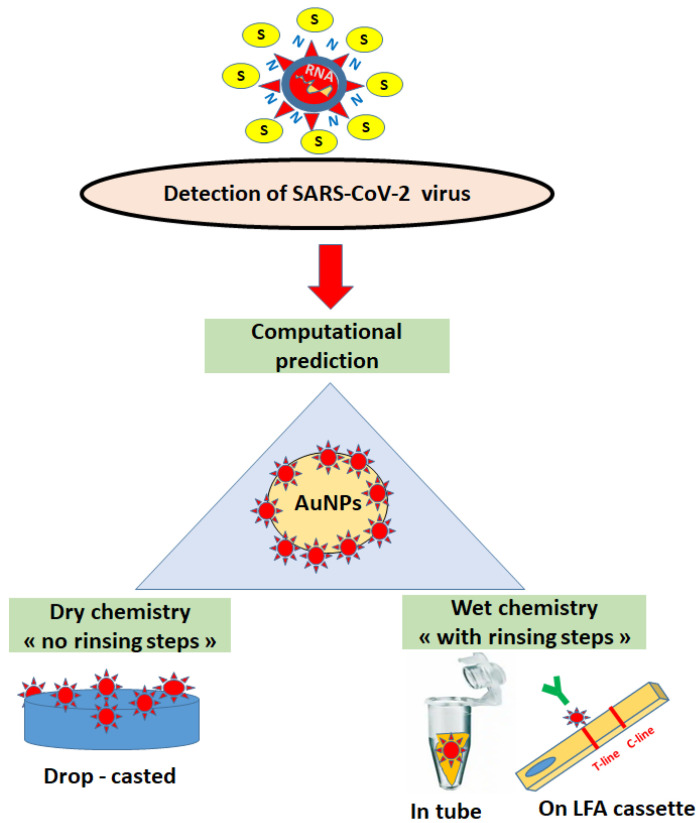
Detection of the SARS-CoV-2 virus and its proteins (S,N) using biofunctionalized gold nanoparticles (AuNPs) for computational prediction and dry/wet chemistry (LFA—lateral flow assay with test (T)-line and control (C)-line).

In the present review, biofunctionalized AuNPs were selected for computational and manual multidetection platforms for the SARS-CoV-2 virus using dry and wet chemistry (Figure 1), either adopting or not adopting buffer washing steps, using the varied biosensing strategies proposed by academic research teams and private markets. In this context, there is still a knowledge gap regarding various computational predictions and patients’ health status, mainly in relation to changes in body fluid composition (viral load, antibody level, antibody types, evolution of blood and urine biomarkers, etc.) in early, middle, and acute SARS-CoV-2 infections. To the author’s knowledge, this review is the first to emphasize the insightful and useful correlations between the theoretical approaches and practicalities of using AuNPs as supports in the construction of multifunctional (portable LFA) platforms for the rapid, sensitive, and selective identification of SARS-CoV-2 in complex biological environments (nasal secretions, saliva, blood, and sera).

## 2. Synthesis of Gold Nanoparticles for In-Tube and On-Paper Medical Diagnoses

**Figure 2:**
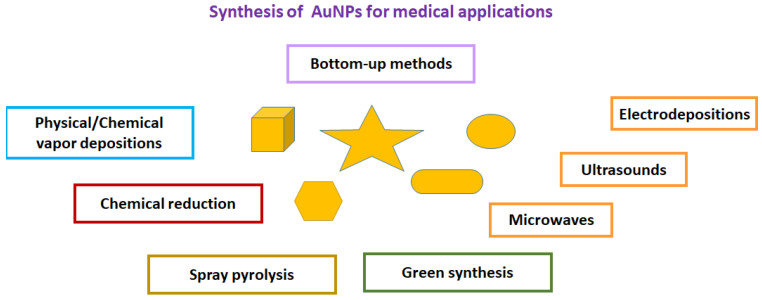
Synthesis of AuNPs using bottom-up methods applicable to the detection of the SARS-CoV-2 virus and its proteins.

Several bottom-up and top-down syntheses of gold nanoparticles continue to be developed, focusing on controlling their size, shape (nanospheres, nanorods, nanocubes, nanoshells, nananostars) [76], composition, and surface modification, for the detection of SARS-CoV-2 virus. Although the top-down methods (e.g., electron-/ion-beam lithography) provide nanoparticles of various shapes and sizes, with high resolution, good stability, and repeatability, they require expensive instrumentation and longer manufacturing times [77,78]. To overcome these limitations, the nanoparticles used for medical diagnoses are commonly prepared with a bottom-up approach, mainly using gas-phase synthesis (e.g., spray pyrolysis) and liquid-phase formation (e.g., chemical reduction) [79] (Figure 2). To date, the water-dispersed colloidal gold nanoparticles (cAuNPs) are synthesized from the metallic precursor HAuCl_4_ in the presence of the chemical sodium citrate, with a color change from colorless to red wine [80], This technique is widely used for rapid LFA [81] tests, even though the fluorescence-dye-labeled immunochromatographic [82,83] method exhibits higher sensitivity and selectivity. Elsewhere, positively charged cysteamine-AuNPs have also been prepared for carrying out specific immunoconjugation events [84].

Studies focusing on the direct synthesis and growth of AuNPs on solid wafers, on nanocellulose paper after microwave irradiation, and on PDMS–glass substrates have used sodium citrate/L-lysine [85], sodium citrate/Na_3_Cl [86], and cetyltrimethylammoniumchloride (CTAB)/ascorbic acid [87] as reducing/capping agent couples. The obtained gold nanostructures were tested as sensitive microfluidic platforms, as SERS substrates for SARS-CoV-2 S protein detection (dried 2 μL, 2 μg/mL, 5 ng/mL), and as ordered arrays of plasmon resonances.

## 3. Computational Predictions of COVID-19 Infection Using Functionalized Gold Nanoparticles 

The development of biosensors based on trial-and-error assays is time consuming when the performances depend on different parameters, including: (i) the stable, fast, reproducible, and reversible interaction of the probe with the target at the microscopic scale, (ii) the size and shape of nanoparticles at the microscopic and mesoscopic scales, and (iii) the optimal experimental conditions for specific targets. 

In the present review, three categories of computations using AuNPs for the significant improvement of plasmonic resonances were proposed for the sensitive detection of the COVID-19 virus. 

The first category focuses on the probe–target interaction by evaluating its strength, velocity, and configuration, as well as the impact on the response of the biosensor. This type of modeling ensures that the chosen probe family is compatible with effective target detection, but it does not guarantee that the performance of the complete biosensor will be satisfactory.

The second category is based on machine learning, one of the methods of which is deep learning. It allows for convergence on an optimal solution, e.g., a probe to detect a given target or a therapy to treat an infection, based on a large amount of data. This type of model is dependent on the quality of the available data. 

Lastly, the third category deals with analytical or parametric models for the optimization of the biosensor configuration (e.g., dimensions, the choice of metals comprising the nanoparticles), from the physical laws characterizing the interaction, the physical properties of materials and interfaces (e.g., the refractive index, permittivity, conduction) and the expected performances (e.g., the wavelength shift, transmitted or reflected intensity). The use of these computations requires the accurate determination of the properties of the materials, which can be difficult in the case of highly heterogeneous media and media of very small sizes. Such properties are often dependent on the experimental conditions.

To summarize, numerous parameters taken into account in the above models aim to improve the resolution of the associated equations, producing more realistic assumptions and neglecting those that only intervene in the second order. Moreover, the selection of the physical law is essential. For example, one study simulated the optical response of different configurations of functionalized AuNPs in the presence of SARS-CoV-2 virions and evaluated the behavior of colloidal solutions containing 105–1013 virions/mL [88]. Interestingly, an equilibration time of 0.3 μs was predicted for peptides (12 derived from ACE2 receptors [89]) to coat the AuNPs (3 nm, 6 nm, and 10 nm) to avoid their aggregation. The resulted peptides-thiol/AuNPs were used for the colorimetric detection of RBD fragments of SARS-CoV-2 S protein. The existence of a strong binding energy between RBD fragments and angiotensin-converting enzyme 2 (ACE2) peptides (66 kcal/mol) was also noted when the peptide-AuNPs were held at a distance of ~2 nm from the spike protein simulated in an aqueous solution [90].

Simulation methods using post-SELEX truncation and mutation were also employed for the evaluation of affinity and the activity characterization of aptamer–target complexes [91]. Moreover, numerical simulations showed that one to three layer(s) of graphene on Ag-Au NPs (15 nm) alloy films should be applied on optical fibers before functionalization with thiol–DNA for transmission measurements in the absence/presence of SARS-CoV-2 RNA, with good sensitivity and resolution [92]. 

Recent study assumed that 152 AuNPs of 10 nm would partially cover the surface of the SARS-CoV-2 virus and could be used for plasmonic detection in the visible range [93,94]. In contrast, another theoretical study reported an optimal viral extinction detection in the mid-infrared range, since a negligible contribution from the surrounding media was noticed [95]. Furthermore, a fully atomistic molecular dynamic (MD) simulation was applied to AuNPs (2 nm) functionalized with peptides in a physiological solution (150 mM NaCl) as sensitive inhibitory agents against SARS-CoV-2 infection; it proved to have a better affinity for RBD than ACE2 [96]. 

Finally, the effects of 100 anti-SARS-CoV-2 drug candidates were systematically evaluated using mathematical algorithms [97].

## 4. Biofunctionalization of Supports and Viral Detection in Spiked Real Samples 

### 4.1. Using Dry Chemistry Functionalization through the Drop-Cast Approach without Rinsing Steps

**Figure 3:**
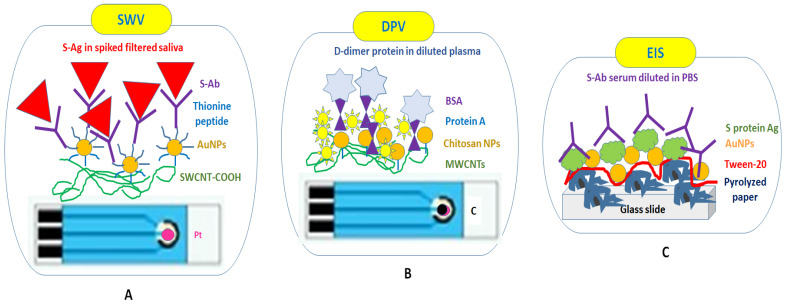
Electrochemical detection of SARS-CoV-2 S proteins and D-dimer with drop-casted biofunctionalized AuNPs using dry chemistry on screen-printed electrodes (**A**,**B**) and Pt/glass slides (**C**). SWV—square-wave voltammetry [99]; DPV—differential pulse voltammetry [100]; EIS—Faradic electrochemical impedance spectroscopy [101].

Drop deposition is commonly used in electrochemical sensing techniques (Figure 3). However, the presence of the “coffee ring” effect can alter the uniform distribution of the nanoparticles that are drop-casted on an electrode. Fortunately, some solutions are proposed to overcome such drawbacks, including the Marangoni effect, anisotropic particles or optimal surfactants [98].

One study reported the use of screen-printed electrodes (SPEs) with platinum (Pt) as a working electrode (WE) for functionalization with carboxyl-modified single-walled carbon nanotubes (SWCNTs-COOH)/peptide thionine (Th)-coated AuNPs/SARS-CoV-2 monoclonal S Ab for square-wave voltammetry (SWV) measurements in the presence of different concentrations of S proteins in PBS and filtered human saliva. When constructing this biosensor, no washing step was performed; therefore, excess bioreagents were not removed from the electrode, possibly affecting the specificity of the immunorecognition events between the S proteins solution and the dried Ab not covalently attached to AuNPs. No measurements using saliva from patients infected with SARS-CoV-2 are reported [99]. SPEs with carbon WE were also modified with multi-walled carbon nanotubes (MWCNTs-COOH) (Table 1) and used for the drop-casting of different bioreagents until complete drying, as follows: chitosan nanoparticles/protein A/D-dimer antibody/bovine serum albumin (BSA)/D-dimer antigen (Ag) present in the diluted plasma of three patients. This rapid and disposable differential pulse voltammetry (DPV) test for D-dimer, a predictive marker of thromboembolic events, was proposed for patients with both thromboembolic and COVID-19 symptoms to improve their medical treatment [100]. It was found that high levels of D-dimer (˃850 μg/L) should not be used for the diagnosis of COVID-19, only for prognosis [100].

Interestingly, pyrolyzed paper was fixed on platinum electrodes patterned on glass slides and served as a porous working electrode for the drop-casting of Tween 20, an irreversible hydrophilic layer against biological fouling/AuNPs/recombinant spike (S) protein. After systematic drying between steps, this biosensor was used for the electrochemical impedance spectroscopy (EIS) detection of SARS-CoV-2 Ab in patient serum diluted with PBS buffer [101].

**Table 1:** Detection of COVID-19 using electrochemical methods and dried layers of drop-casted biomolecules without rinsing steps (*—target entity).

			Drop Casting				
Support	Method	Antibodies (Ab)	Antigens (Ag)	LOD	Advantages	Disadvantages	Ref
SPE-Pt	SWV	Monoclonal anti-S Ab	S protein *	200 pM in PBS 500 pM in filtered saliva	Presence of SWCNTs	Dried Ab Short incubation time (5 min) between Ab and Ag No rinsing	[99]
SPE-C	DPV	D-dimer Ab	D-dimer *	0.6 μg/L in PBS	Presence of MWCNTs and D-dimer, predictive markers of thromboembolic events important for viral infections Optimized incubation time (15 min)	Dried Ab No rinsing	[100]
Pyrolized paper-Pt	EIS	Ab in serum diluted with PBS *	S protein	NA	Presence of Tween-20	Dried Ab No washing steps	[101]

### 4.2. Using Wet Chemistry for Biofunctionalization in Eppendorf Tubes, including Centrifugation and Rinsing Steps

**Figure 4:**
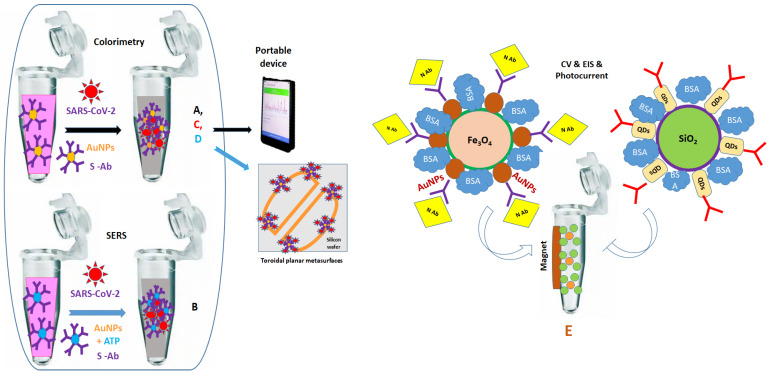
Optical and electrochemical detection of SARS-CoV-2 S and N-proteins using biofunctionalized gold nanoparticles (AuNPs) and wet chemistry “in tube” with (**A**,**B**), [102], (**C**), [103], (**D**), [104], and (**E**), [105]; SERS—surface-enhanced Raman spectroscopy; CV—cyclic voltammetry; EIS—electrochemical impedance spectroscopy.

Colorimetric and SERS measurements are proposed for the rapid (five min) detection of S proteins and SARS-CoV-2 pseudo-virus, using Eppendorf tubes and glass slides as supports for recording optical spectra from a few mL and dried drops of (bio)reagents, respectively (Table 2). For colorimetric assays, aqueous-citrate-coated AuNPs modified with carboxy-PEG-thiol (HS-PEG-COOH) were biofunctionalized with anti-S Ab, developing a pink color visible to the naked eye, which turned blue due to aggregation in the presence of specific antigens, namely, S protein and pseudo virus particles. For the SERS studies, AuNPs modified with 4-aminothiophenol (ATP) were functionalized with anti-S Ab and used for the detection of S protein and pseudo viral particles. In this work, the authors found that AuNPs functionalized with 100 ng/mL of anti-S Ab blocked 100% the viral replication by disrupting the lipid membrane of the SARS-CoV-2 pseudo virus, causing its collapse and preventing it from entering living cells (Figure 4A,B) [102].

Additionally, inactivated SARS-CoV-2 virus spiked in PBS buffer, water rivers, and artificial and human saliva was optically detected in Eppendorf tubes with a smartphone camera and an adapted interface that allowed for the visualization of significant color changes due to the functionalized AuNPs with MUA/SH-PEG/anti-S polyclonal Ab/BSA. Moreover, the contamination of São Carlos water samples spiked with inactivated SARS-CoV-2 virus was optically confirmed using the portable device (Figure 4C) [103]. 

Colloidal HS-AuNPs conjugated in Eppendorf tubes with anti-S Ab and three concentrations of the SARS-CoV-2 S Ag were dried on gold-coated toroidal planar metasurfaces prepared on silicon wafers. As expected, the AuNPs that dried on the Au films strongly confined the electromagnetic fields, ensuring significant frequency shifts for different antigen concentrations and rinsing steps with PBS. However, these sensitive metastructures have not yet been tested in the presence of real samples (Figure 4D) [104].

A magnetically assisted photoelectrochemical immunosensor (PEC) was constructed to detect the N protein of SARS-CoV-2 using Eppendorf tubes for the magnetic separation and washing steps before the electrochemical (CV, EIS) and optical (photocurrent in the nA range) investigations. Two main synthetic routes were proposed: one for the synthesis of Fe_2_O_3_/SiO_2_/AuNPs (called M-AuNPs) and the other for SiO_2_/TiO_2_/CdQDs (called P-QDs). The M-AuNPs were then used for conjugation with anti-N antibody 2D3 (Ab1)/SARS-CoV-2 N protein (Ag), while the P-QSs were used for conjugation with anti-N antibody 3F2 (Ab2). Finally, the biofunctionalized M-AuNPs/Ab1/SARS-CoV2-N and P-QS/Ab2 nanoparticles were mixed. After the PBS washing step, drops of the sandwich-form immune complexes were deposited on ITO electrodes and dried before the photocurrent measurements. As expected, the higher the concentration of the SARS-CoV-2-N protein, the more P-QS particles were combined with M-AuNPs, inducing higher photoelectric response values specific for an increased number of immune complexes (Figure 4E) [105].

### 4.3. Using Wet Chemistry for the Biofunctionalization of Solid Substrates, including Rinsing Steps 

**Figure 5:**
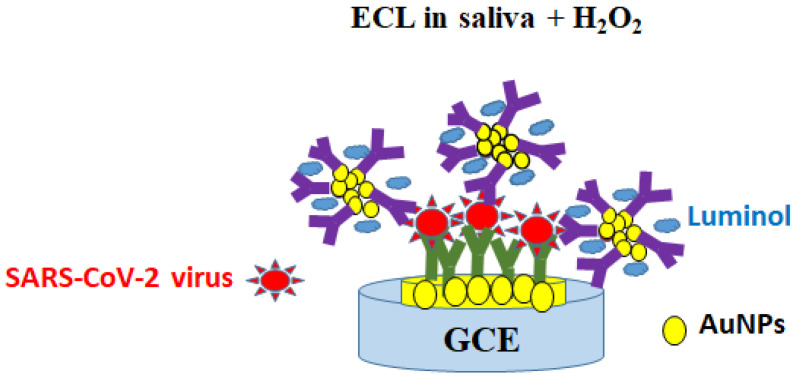
Optical detection of SARS-CoV-2 S proteins using biofunctionalized AuNPs and wet chemistry on solid electrodes with rinsing steps. ECL—electrochemiluminescence.

A sandwiched electrochemiluminescence (ECL) immunosensor using two antibodies for the detection of inactivated SARS-CoV-2 virus in PBS in about two hours was reported after two days of a functionalization protocol. The electrodeposition of flower-like AuNPs (60 s) on GCE was followed by the immobilization of Ab1 (polyclonal anti-SARS-CoV-2 Ab in rabbit, 13 μg/mL/SARS-CoV-2 inactivated virus vaccine (Coronavac–Sinovac, 1 mg/mL) and tested using luminol-colloidal AuNPs@Ab2 (the polyclonal anti-SARS-CoV S Ab in goat which identifies the S protein, 1.8 mg/mL). A photomultiplier tube (PMT) and smartphone were used to record the ECL signals related to the formed immunocomplexes, which were validated using saliva as a clinical sample collected from five infected positive and five healthy negative patients (Figure 5) [106].

AuNPs were functionalized with recombinant G protein (G-cAuNPs) and used for the detection of anti-SARS-CoV-2 IgG Ab in the presence of the full-length SARS-CoV-2 spike proteins that were immobilized on homemade eight-well gold interdigitated electrode (IDE) chips. In the case of positive serum samples, the impedance of IDE was altered by the G-cAuNPs, while, for the negative serum samples, the impedance of IDE remained the same since no IgG or G-cAuNPs were bound to the IDE surface [107].

**Table 2:** Detection of COVID-19 using optical/electrochemical/frequency methods and wet chemistry in tubes with rinsing steps (*—target entity).

			Tubes/Surfaces				
Support	Method	Antibodies (Ab)	Antigens (Ag)	LOD	Advantages	Disadvantages	Ref
Tubes and glass slide	Colorime-tric and SERS	Monoclonal anti-S Ab	S protein * 1 ng/mL; 4 pg/mL and SARS-CoV-2 * Pseudo-virus 1000 particles/mL: 18 particles/mL	NA	Rapid test (5 min) Presence of 100 ng/mL anti-spike Ab blocked 100% of viral replication	Dried Ag for SERS	[102]
Tubes	Colorime-tric	Polyclonal Ab	S protein * and inactivated SARS-CoV-2 * virus in PBS, water river, artificial saliva, human saliva	2.2 PFU/mL PBS and 0.28 PFU/mL human saliva and 7; 250; 1000; 5000; 6000 PFU mL	Covalent functionalization of AuNPs with MUA and thiol-PEG Optical signal reading with smartphone	Use of polyclonal Ab	[103]
Tubes and toroidal planar metasurfaces	Frequency shifts	Monoclonal anti-S Ab	S protein * in PBS (4 fM, 8 fM, 12 fM)	NA	Use of colloidal thiol-AuNPs	Dried Ag on AuNPs No tests with real samples	[104]
Tubes and ITO	Photo- current	Two sources of anti-N Ab	N protein * (1 h)	1.8 pg/mL	Easy NPs washing by holding magnet on tubes	Multiple steps for NPs synthesis	[105]
GCE	ECL	Two sources of polyclonal anti-SARS-CoV-2 Ab	Inactivated SARS-CoV-2 virus * in PBS (10 ng–10 μg/mL) (2 h)	1.93 ng/mL	Use of active probes (luminol) Optical signal reading with smartphone	Time-consuming protocol (2 days)	[106]
IDE	Impedance	SARS-CoV-2 IgG Ab in 10 × serum * (4 μL)	S protein (0.1 mg/mL)	NA	Easy monitoring of positive virus with change in frequency	Multistep preparation of IDE supports	[107]

### 4.4. Using Lateral Flow Wet Chemistry for the Biofunctionalization of Metallic and Flexible Paper-Based Substrates including Rinsing Steps

**Figure 6:**
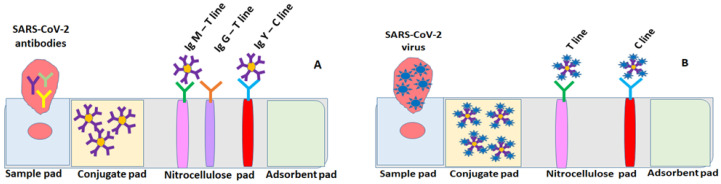
Different configurations of LFA cassettes for screening for the presence of SARS-CoV-2 in nasal swabs/serum/plasma/saliva samples (**B**) and the production of its specific IgA/IgM/IgG Ab (**A**) using biofunctionalized gold nanoparticles (AuNPs) and wet chemistry “on-paper” support electrodes with rinsing steps for colorimetric, SERS, and photothermal rapid testing.

Flexible nitrocellulose microporous membranes are used in the construction of the immunochromatography tests known as lateral flow assays for the qualitative visualization of immunocomplexes formed in the presence of antigens/antibodies of interest in various bodily fluids (nasal swabs, whole blood, serum, saliva, urine). For general public use or for more advanced medical investigations, LFAs are widely recommended due to their simplicity, single-use nature, cost effectiveness, and portability. These assays mainly consist of a sample pad, a conjugate pad, an NC membrane, and an absorbent pad, which are all packed in a polystyrene cassette for easier handling (Figure 6). When running the test, a sample droplet migrates from the conjugate pad to the absorbent pad due to capillary forces within about 15 min. In this section, LFA generations are discussed.

Conventional LFAs employ a citrate buffer for the stabilization of colloidal gold nanoparticles (cAuNPs), while more recently prepared gap-enhanced Raman polygonal gold nanotags (Au-GNPs) have been proposed as ultrasensitive methods of generating colorimetric and SERS signals. As mentioned above, the target analyte and Ab-labelled AuNPs will form immunocomplexes and bind to the detected antibodies line (the T (test)-line) when a positive signal appears as a red line. However, the main drawback of the LFA approach is the T line’s intensity, which is not always visible to the naked eye and is not always distinct from the background; therefore, false negatives are commonly obtained due to very low target concentrations, generating misleading LFA results. However, for optimal sample testing, the remaining Ab-AuNPs that do not react with the target should bind to the secondary antibodies in the control (C) line and form a red line, which should always appear in the absence or presence of the assessed antigen. Thus, LFAs often suffer from lower sensitivity [108] and require improvements to decrease the risk of viral contamination [109].

In this section, we discuss the latest advances in the fabrication and use of LFAs for the traditional detection of the SARS-CoV-2 virus and its proteins, as well as the first attempts to use LFA detection of the specifically designed synthetic DNA sequence target for the N region of SARS-CoV-2 in real human samples. 

#### 4.4.1. Conventional Protein Antigen/Antibody-Based LFAs: First Generation

##### Detection of SARS-CoV-2 N protein using nasal swabs

One study reports the use of nasopharyngeal swabs for the detection of SARS-CoV-2 N protein on nitrocellulose (NC) [110]. Before LFA testing, the conjugate pad was modified with dried immunocomplexes made of recombinant anti-SARS-CoV-2 Nucleocapsid antibody (Ab1)-cAuNPs (1:2 ratio). The two fine micro-sprayed lines used as the detection T-line were made of dried Ab1, while the C line was made of dried monoclonal goat anti-mouse IgG (Ab2) (Table 3). 

##### Detection of SARS-CoV-2 RBD Using Nasal/Throat Swabs

The receptor-binding domain of SARS-CoV-2 was selected as the viral antigen, and it was detected in 100 μL PBS buffer and 50 diluted positive samples extracted from the nasopharynx before being added to the LFA strip for a 15 min test. Next, the intensity of the colorimetric signal obtained from the test line was analyzed using a smartphone colorimeter application called ColorGrab. Interestingly, the proposed test showed signal stability over 21 days when stored at 4 °C and no cross-reactivity with the influenza virus. The optimized LFA used a NC membrane modified with anti-RBD antibodies conjugated to cAuNPs for drop-casting on the conjugate pad, an anti-RBD antibody (on T-line) and anti-rabbit IgG antibody (on the C-line) for the detection zone that is visible to the naked eye, and an absorbent pad for the sample flow collection via capillary forces [111].

Recently, the LFA was modified with a trimethylsilyl cellulose barrier added to the CN140 hydrophobic NC membrane before the T-line to slow down the arrival of SARS-CoV-2 S RBD conjugated on cAuNPs. Thus, improvements were recorded in the recognition time between the SARS-CoV-2 S1 IgG and the SARS-CoV-2 S RBD Ag, which continues to accumulate when additional antibodies are present in positive samples. For the C-line, the SARS-CoV-2 S1 IgM antibody was used and validated the LOD of 0.11 ng/mL, 9.1 times lower than the classic LFA. Additionally, eight positive throat swab specimens were confirmed with the new LFA configuration [112]. 

On the other hand, the GeneTex Company proposes 2 LFA antigenic assays for viral detection in approximately 15 min in bodily fluids: one that screens for neutralizing antibodies to the SARS-CoV-2 S (RBD) that develop on the initial T-line modified with the RBD spike protein, and the second for SARS-CoV-2 virus on the initial T-line modified with the anti-S (RBD) antibody. For positive individuals, using the first kit configuration, the T-line modified with the grafted RBD protein is uncolored in the absence of antibodies from the tested sample, while the C-line modified with the grafted anti-ACE2 antibody is colored red in the presence of displayed human ACE2-AuNPs from the conjugation pad. In the second antigenic configuration, the T-line is colored red-purple in the presence of the SARS-CoV-2 virus due to the immunocomplexes formed with the anti-ARS-CoV2 antibody grafted on the paper. Meanwhile, the C-line is also reddish in color, as displaced Ab-AuNPs from the conjugation pad become attached to the secondary Ab grafted on the paper and promote their agglutination.

##### Detection of IgA, IgG, and IgM Antibodies in Serum and Nasal Samples 

Serological tests for anti-SARS-CoV-2 IgA/IgM/IgG are of great importance to reducing the extent of viral contamination. The presence of IgM [113] and IgA has been noted mostly in asymptomatic and mild infections when IgA plays an important role in mucosal immunity, whereas IgG appears >14 days after the onset of symptoms [114].

The rapid LFA detection (15 min) of IgM antibodies (indicators of the viral acute infection period) in positive SARS-CoV-2 serum samples is proposed. In practical terms, conjugates of anti-IgM human antibody-cAuNPs (30 nm) were dropped on a conjugate pad, while SARS-CoV-2 and goat anti-mouse IgG were used for the T-line and C-line, respectively. The prepared LFA strips exhibited selectivity in the presence of thrombocytopenia syndrome virus (SFTSV) and dengue virus (DFV) [115].

A portable LFA nitrocellulose cassette was developed for the detection of human antibodies in serum samples (50 μL), specifically, the immunoglobulins M and G (early IgM and late IgG) were directed against two recombinant proteins, such as the N protein and the RBD of the SARS-CoV-2 S protein. These proteins were conjugated to 30 nm colloidal Au NPs and deposited on the conjugation pad. Goat anti-human IgG and IgM were used to draw the T-lines, while anti-mouse IgG (raised against both recombinant proteins) was used for the C-lines. It should be noted that, for those individuals infected for less than one week, the T- and C-lines on the NC strips were visible to the naked eye through their reddish color change caused by the presence of IgM and IgG of the N and RBD proteins [116]. Clinical studies confirmed the presence of the anti-IgA antibody in positive infected individuals in both early and late-stage SARS-CoV-2 infection [117]. For example, anti-IgM/IgG/IgA were simultaneously LFA detected (15 min) in positive serum/plasma (10 μL) dispensed onto sample pads that displaced the protein A-cAuNP conjugates dried on the fiberglass conjugation pad. The recombinant SARS-CoV-2 N protein and protein A were immobilized on the NC membrane at the T-line and C-line, respectively. Interestingly, the authors found that the visibility of the T- and C-lines were not affected by the presence of anticoagulants such as TSC, Heparin, and EDTA in the spiked negative, weak positive, and strong positive samples. LFA was also assessed for cross-reactivity with other virus types where the T-line did not show any visible response [118].

##### Detection of the SARS-CoV-2 Virus Using Glyco-Based LFA: The Influence of Silver Staining

Glycans are often more thermally robust than proteins; they are directly involved in the pathogen adhesion during viral infections and participate in the binding of the angiotensin-converting enzyme 2 (ACE2) receptor during SARS-CoV-2 cell adhesion/entry [119]. An initial glycan-based LFA lacking a T-line but with directly spotted low-volume samples (collected from 50 positive nasal swabs) was developed using an N-acetyl neuraminic-acid-modified polymer to coat AuNPs and capture the active/inactive SARS-CoV-2 virus via sialic acid from protein S’ binding site. For colorimetric signal enhancement, a silver-staining step was introduced, assuming that the silver ions were reduced to insoluble metallic silver in aqueous solutions with precipitation on the surface of AuNPs [120]. Another LFA study proposed the use of anti-S-antibody biofunctionalized AuNPs for the binding of the SARS-CoV-2 virus in the conjugation pad, followed by migration to a test line made of a biocompatible glycocalyx “sugary”-matrix-based biotin polysaccharide conjugated with streptavidin and rabbit antihuman-IgG for the efficient detection of SARS-CoV-2 S/Ab-AuNPs immune complexes [121].

#### 4.4.2. Pressed Protein Antigen/Antibody LFAs in the Center: Second Generation

A study based on LFA principles proposed the use of a manual pressure point on the central zone of the NC paper strip, between the test (T) line and the control (C) line, to enhance signal intensity in the presence of a specific antigen due to a local reduction in the pore size and an increase in the fluid resistance. Therefore, slowing down the flow of the liquid loaded with trace amounts of an antigen such as CRP protein/SARS-CoV-2 N protein resulted in an increase in the anti-CRP antibody(Ab)-AuNPs or kit Ab-AuNPs/Ag conjugation time and in the number of immunocomplexes formed, which significantly improved the colored signal (T-line) and the detection limit. The authors found the optimal pressing condition at 15.69 MPa for CRP detection in PBS/CRP-free diluted human serum and at 23.54 MPa for detection of the SARS-CoV-2 N protein in two commercial COVID kits, namely, the STANDARD™ Q COVID-19 Ag Home Test and Humasis COVID-19 Ag Home [122].

#### 4.4.3. SERS Optics for Protein Antigen/Antibody LFAs: Third Generation with Dual- and Tri-Mode Detection Signals 

**Figure 7:**
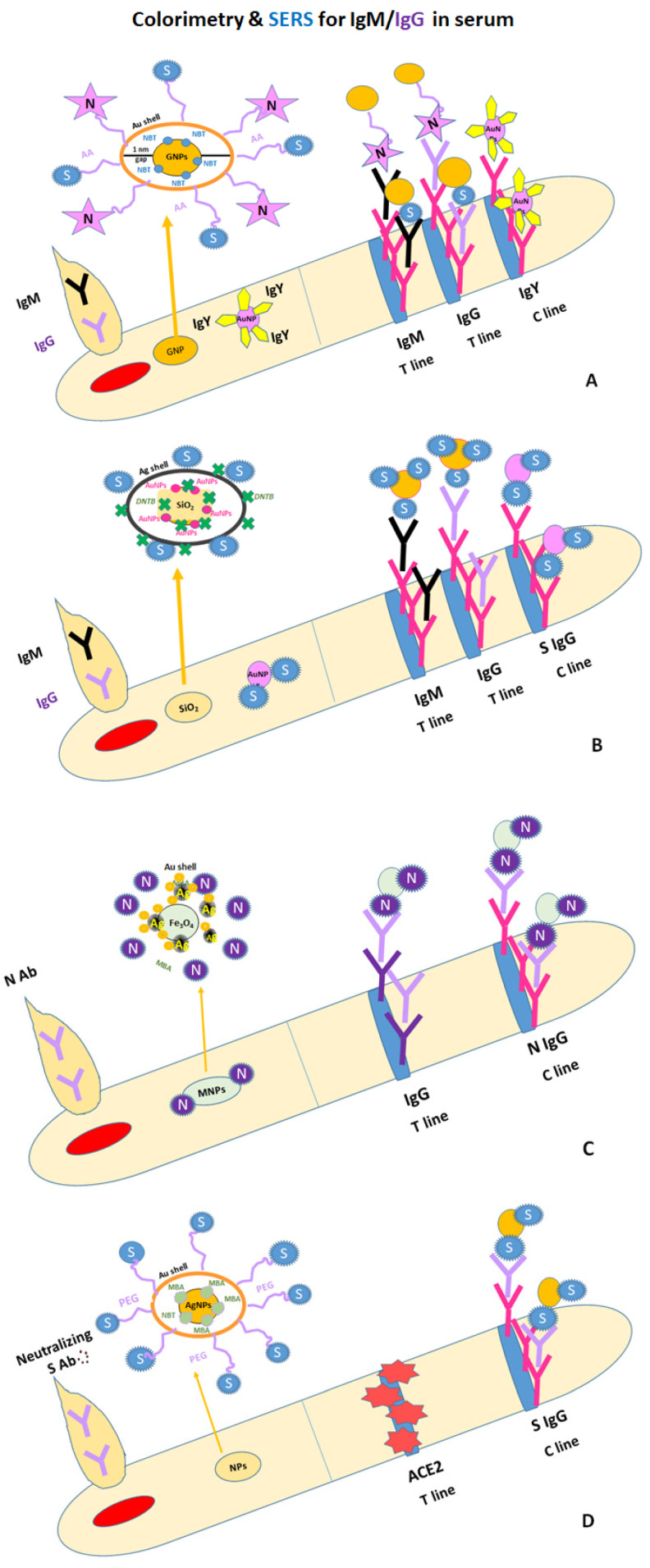
LFAs for simultaneous and individual SARS-CoV-2 IgM and IgG detection in human sera using different coatings on AuNPs and dyes as Raman enhancers: (**A**) one layer of NBT—4-nitrobenzenethiol [123]: (**B**) two layers of DTMB—5,5-dithiobis-(2-nitrobenzoic acid [124]; (**C**,**D**) one layer of MBA—4-mercaptobenzoic acid [125,127].

Recently, four studies [123,124,125,126] have proposed the use of Raman molecules in the functionalization of AuNPs as enhancers of colorimetric and SERS signals on one/two T-line LFA strips for single anti-SARS-CoV-2 N/S antibodies or simultaneous IgM/IgG detections (Table 3). Thus, the first study reports the use of gap-enhanced Raman polygonal gold nanotags (Au-GNPs), which are biofunctionalized with recombinant COVID-19 antigens (S-protein/N-protein), for the instantaneous detection of IgM/IgG in positive blood/serum samples. The homemade LFA strips had two T-lines (IgM/IgG) that were analyzed using rapid naked-eye colorimetric evaluation and advanced microscopic surface-enhanced Raman spectroscopy (SERS) measurements; they were more accurate than colorimetric data and had 100-fold the sensitivity. For the preparation of the Au-GNPs, the adsorption of 4-nitrobenzenethiol (4-NBT) on the Au’s core surface over 30 min was an obligate spacer molecule for the formation of a 1 mm gap between the core and the shell. Then, the C line was coated with polyclonal goat anti-chicken IgY Ab to capture the AuNPs [123] (Figure 7A).

In a second study, the simultaneous detection of anti-SARS-CoV-2 IgM/IgG in 19 positive clinical samples was reported using a naked eye colorimetric visualization and a portable Raman system for SERS signals on an LFA strip. To improve the SERS signal, the authors prepared nanoparticles labelled with dual layers of Raman dye (5,5-dithiobis-(2-nitrobenzoic acid—DTNB), namely, SiO_2_ (200 nm)/AuNPs (3 nm) and DTNBs (1), followed by coating with AgNPs(3 nm) and DTNBs (2). The obtained nanocomposites were biofunctionalized with a SARS-CoV-2 S protein that conjugated with anti-SARS-CoV-2 IgM/IgG from positive samples in the conjugation pad, which moved on the NC membrane to the IgM T-line, the IgG-T line, and the C-line, respectively [124] (Figure 7B). 

**Table 3:** Detection of COVID-19 using optical/electrochemical/frequency methods and four generations of LFA tests (*—target entity).

			First LFA				
Paper Support	Method	Bioreagents on AuNPs	Antigen (Ag)	Bioreagents on T-Line/C-Line	Advantages	Disadvantages	Ref
NC	Colorimetric	Anti-N 20 ng/mL (minimal concentration)/cAuNPs (1:2)	N-protein * (2.14 ng/mL) on nasal swabs	Ab_1_ 2 mg/mL and Ab_2_ 2 mg/mL	Low concentrations and volumes of Ab for conjugation on AuNPs	High optimal concentration of Ab on T/C-lines Local optimization	[110]
NC (12 μ pore size)	Colorimetric	Anti- RBD (1 mg/mL)/ cAuNPs (~15 nm, pH 9)	RBD * on nasal swabs in PBS and spiked PBS (LOD 1 ng/mL)	Ab_1_ 1 mg/mL and Ab_2_ 1 mg/mL	100 μL sample T-line readings with smartphone Signal stability for 21 days	Local optimization	[111]
NC CN140	Colorimetric	RBD (1 mg/mL in PBS):cAuNPs (~20 nm, pH 9)	RBD * in PBS LOD 0.11 ng/mL RBD * in throat swabs	S-IgG (500 μL/Ml in PBS) on T-spot and IgM (1 mg/mL in PBS) on C-spot	Presence of trimethylsilyl cellulose barrier and upper extra sample pad layer before T-line to slow down the flow Improved LOD Target detected on T-spot with no line	Lack of robustness of local optimization: Spacing between barrier/T spot/C spot Homogeneity of spot size	[112]
NC + 1 mg/mL BSA	Colorimetric	IgM human (1.5 μg)-cAuNPs (30 nm)	SARS-CoV-2 Ig M * in serum samples (20 μL/80 μL running buffer PBS/BSA/Triton)	SARS-CoV-2 (1 mg/mL) on T-line and goat anti-mouse IgG (2 mg/mL) on C-line	Good selectivity in the presence of other viruses 10x test/sample very low sample volume 10–20 μL)	Laborious strip optimization	[115]
NC	Colorimetric	N (70–80 μg/mL) /Au NPs (30 nm) and RBD (60–70 μg/mL)/AuNPs (30 nm) pH 9	Simultaneous IgM * and IgG * in serum samples	On T-line: goat anti-human IgG (1 mg/mL) and IgM (1.2 mg/mL) On C-line: anti-mouse IgG 2 mg/mL for N and 1.8 mg/mL for RBD	Early detection of SARS-CoV-2 infection (<1 week) Visible T-line	No calibration curve No LOD	[116]
NC	Colorimetric	Protein A-cAuNP (pH 8.5–9)	Simultaneous IgM */IgG */IgA * in serum/plasma	N protein (0.9 μg/cm) on T-line and Protein A (0.3 μg/cm) on C-line	No influence from anticoagulants on the color of T-/C-lines No cross-reactivity with other viruses	No calibration curve No LOD	[118]
NC Immunopore	Colorimetric	Glycan-AuNPs (35 nm)	Inactivated SARS-CoV-2 * collected from nasal swabs	Spot of SARS-CoV-2 S protein-bearing lentivirus and Lectine on C-line	Sample spot instead of T-line Very low sample volume (2 μL) Improved color visibility due to silver staining step	Need for polymeric coating to reduce nonspecific interaction	[120]
NC	Colorimetric and enhanced signal in the presence of HRP/AEC reagents	Anti-S Ab/AuNPs (10 nm)	SARS-CoV-2 S * in saliva Visible to naked eye for: HEP at 3.13 μg/mL in 25 μL, and on HS at 3.13 μg/mL in 50 μL	On T-line: GAG with streptavidin (1 mg/mL) and on C-line: Anti IgG (1 mg/mL)	Signal stability over 47 days at RT Computation data LOD (0.78 μg/mL in 25 μL) 4-fold lower compared with unamplified results	Laborious strip optimization	[121]
			**Second LFA**				
NC 10 μm pore size (CNPF-SN12)	Colorimetric	Anti-CRP (2 mg/mL)/ AuNPs (20 nm)	CRP protein * in PBS and serum and COVID-19 Ag kit 2-fold increased signal	Anti-CRP Ab (1 mg/mL) on T-line and goat anti-mouse IgG Ab on C-line	Pressure zone between T-line and C-line induces flow delay and signal is enhanced	No tests with samples from SARS-CoV-2 infected patients	[122]
			**Third LFA**				
NC 8 μm pore size (CN140)	Colorimetric and SERS	S-protein/ N-protéiné on Au-GNPs (10 μg/ 1.1 × 10^−3^ g GNPs (55 nm) + 4-NBT	Simultaneous IgM */IgG * in positive blood/serum samples	Mouse anti-human IgM (1 mg/mL)/ mouse anti-human IgG (1 mg/mL) on two T-lines and polyclonal goat anti-chicken IgY antibody (0.5 mg/mL) on C-line	Two T lines 4-NBT (5 μg/mL) on AuGNPs 100 times more sensitive than colorimetric result LODs: 1 ng/mL for IgM and 0.1 ng/mL for IgG	Instability of 4-NBT on AuNPs No data on storage of Au-GNPs /buffer/tempe-rature	[123]
NC ~10 μm pore size (CN140)	Colorimetric and SERS	SARS-CoV-2 S/ nanocomposites (30 μg of S protein/ mL of SERS tags) + DTNB	simultaneous IgM */IgG * in 10^3^×, 10^4^×, 10^5^×, 10^6^× diluted serum and 1% whole blood samples after 25 min immunoreaction	Goat anti-human IgM (0.5 mg/mL)/ goat anti-human IgG (0.6 mg/mL) on two T-lines and SARS-CoV-2 S Ab (0.5 mg/mL) on C-line	DTNB Raman dye SERS signal at 1328 cm^–1^ for IgM andIgG 800 times more sensitive that LFA based on AuNPs LOD_SERS_ 1 pg/mL	No SERS data on individual components of blood/serum samples for patients with different pathologies	[124]
NC	Colorimetric and SERS	SARS-CoV-2 N (10 μg/mL)/ trimetallic hybrid MNPs + MBA	anti-SARS-CoV-2 N Ab * in positive serum samples	Mouse anti-SARS-CoV-2 N Ab (1 mg/mL) on T-line and rabbit anti-human Ab (1 mg/mL) on C-line	LOD 10^−8^ mg/mL MBA Raman dye SERS signal at 1075 cm^−1^, linear within the range of 10^−10^ to 10^−6^ mg /mL Ab * LOD 0.08 pg/mL	No data on the stability of hybrid MNPs with MBA over time and influence of washing, RT and 4 °C in SERS signal evolution	[127]
NC CN140	Colorimetric and Photothermal and SERS	SARS-CoV-2 S protein (10 μg/mL)/ bimetallic Au–Ag HNSs + MBA	Neutralizing SARS-CoV-2 Ab * in spiked serum samples 10x diluted with PBS (20 to 1500 ng/mL) and blood	ACE2 protein on T-line and SARS-CoV-2 S protein Ab on C-line	MBA Raman dye LOD 160 ng/mL LOD 20 ng/mL LOD 20 ng/mL	Missing the SERS signal information used for the calibration curve No data on the stability of NPs with MBA over time and influence of washing, RT and 4 °C in SERS signal evolution	[125]
			**Fourth LFA**				
NC M170	Colorimetric	Thiol–DNA probe (4 and 8 μM)/ AuNPs	SARS-CoV-2 N gene *	SARS-CoV-2 N gene complementary on T-line and complementary DNA probe on C-line	3 sandwich nLFA models 50 bp length of the target influence LOD 5 pM in SSC buffer	Laborious explanation Manually micro- pipetting lines No calibration curves for the three proposed nLFA models	[126]
NC HF120	Colorimetric	Thiol–DNA detection probe/AuNPs (~13 nm) (7 μL)	SARS-CoV-2 RNA *	Biotin–DNA capture probe (0.5 μL of 25 μM) on T-line and biotin–DNA control probe (0.5 μL of 25 μM) on C-line	-Two sugar barriers (10 %) before (2.5 μL) and after (1 μL) the T-line to slow down the flow and improve the test sensitivity by 5-fold	No data on storage and signal stability No data using positive samples	[128]
NC	Colorimetric and digital camera and SERS (laser 532 nm)	Two antisense oligonucleotides ASO (25 μM) labeled with FAM (30 μM) and biotin (30 μM) Optimization: ASO_1_-biotin ASO_2_-FAM	Inactivated SARS-CoV-2 N gene * (RNA/cDNA) in 10x diluted swab sample and 67250; 3362; 168; 8; 0.42; 0.02; 0.001 copies/μL	Anti-FAM Ab + Av-AuNPs on T-line and anti-Av Ab on C-line	30 min test after collection of swab Cys-AuNPs as signal enhancer of T-line color for concentrations below 8 copies/μL LOD of 0.02 copies/μL SERS signal at 1617 cm^−1^ strongly increased Validated in 30 positive nasal/nasopharyngeal samples	Naked-eye color evaluation not possible until 8 copies/μL No calibration curve based on SERS signals Several parameters require optimizations by end users	[129]
NC	Colorimetric and smartphone	Anti-FITC Ab/AuNP	SARS-CoV-2 N gene detection in cDNA and SARS-CoV-2 RNA * from swabs tested positive with real-time qRT-PCR (5.6 × 10^6^ to 3.9 × 10^3^ copies/mL)	Streptavidin on T-line and anti-goat IgG Ab on C-line	5 μL of LAMP-amplified product based on biotin-dUTP/FITC-LF + 20 μL running buffer LOD 3.9 × 10^3^ RNA copies/mL	Laborious protocol Temperature ˃60 °C for active polymerase enzyme and not applicable for kit market	[130]
NC	Colorimetric	Anti-FAM Ab/AuNPs	RBD * within the S gene of SARS-CoV-2, (synthetic DNA, 16.7 nM)	SA on T-line and anti-rabbit Ab on C-line	Biotin primer for easy linking to SA-NC FAM probe	Costly protocol No data with real positive samples	[131]

In a third study, colorimetric and SERS dual-LFA (dLFA) were also successfully performed for the quantification of anti-SARS-CoV-2 N Ab in positive serum samples (from 9 unvaccinated and 98 vaccinated volunteers). This study used trimetallic hybrid (MNPs) nanomaterials consisting of magnetic nanoparticles (Fe_2_O_4_NPs) that were electrostatically coated with silver/4-mercaptobenzoic acid (MBA-ERS probe) and AuNPs and biofunctionalized with the SARS-CoV-2 N protein. The resulting N-protein/MNPs were incubated/mixed with positive samples containing nucleocapsid antibodies using the Eppendorf wet chemistry approach; this was followed by magnetic separation and purification (p). Finally, pN-Ab/MNPs were dropped on an LFA strip; they then moved to a T-line made of rabbit anti-human IgG antibodies (the color changed to red), while excess immunocomplexes became bound to the C-line, which was made of mouse anti-N-protein antibody (color also changed to red), thus validating the LFA test [127] (Figure 7C).

For the more sensitive and accurate quantification of SARS-CoV-2-neutralizing antibodies, a three-mode competitive LFIA (tLFA) based on colorimetric, photothermal, and SERS detections was developed using hybrid bimetallic nanoparticles based on gold–silver alloy hollow nanoshells (Au/AgNPs) modified with 4-mercaptobenzoic acid (MBA) for the enhancement of SERS signals. This tLFA, which detects neutralizing antibodies against SARS-CoV-2, evaluated the ability of Ab to inhibit the binding between the SARS-CoV-2 S protein RBD and the angiotensin-converting enzyme 2 (ACE2). Therefore, no color was noticed on the T-line sprayed with the ACE2 protein, whereas a red color was visible on the C-line sprayed with SARS-CoV-2 S protein IgG for 79 out of 98 vaccinated individuals [125] (Figure 7D).

#### 4.4.4. Nucleic Acid LFAs: Fourth Generation

An initial nucleic sandwich assay (nLFA) [126] for the detection of synthetic target DNA (50 bases long) specific for the N gene of SARS-CoV-2 in a saline-sodium citrate (SSC) running buffer was reported. The nLFA used two concentrations of thiol–DNA probes (4 and 8 μM) for immobilization on cAuNPs, while streptavidin-modified biotin-oligonucleotides were micro-pipetted directly onto the T- and C-lines (Table 3). 

One nLFA prototype proposed the addition of two sugar barriers (drops) before and after the T-line, resulting in the five-fold improved sensitivity of SARS-CoV-2 RNA (44 bp) detection due to an increased reaction time with the 5’thiol-detecting DNA probe cAuNP and further with the biotin–DNA capture probe (20 bp) on the T-line [128].

Aggregates of cysteamine-AuNPs/streptavidin-AuNPs were used as nano-amplifiers of colorimetric/SERS LFA signals formed in the presence of extracted target SARS-CoV-2 RNA from 30 positive nasal/nasopharyngeal swab samples (30 min test) and two specific 6-carboxyfluorescein (FAM)/biotin-labeled antisense DNA oligonucleotide probes (20 bp). The anti-FAM antibody and anti-streptavidin antibody were used for the T-line and C-line, respectively [129].

Furthermore, the loop-mediated isothermal amplification (LAMP) technique for specific nucleic acid amplification was combined with the fLFA for SARS-CoV-2 N gene detection in 82 cDNA and clinical swab-extracted RNA samples. The nLFA was analyzed with a smartphone interface; a streptavidin (SA) printed T-line appeared with a red color only when the LAMP-amplified product based on biotin-dUTP/FITC-LF was detected by the AuNP anti-FITC antibody. Unfortunately, the implementation of LAMP for commercial kits is not yet possible due to the higher temperatures (˃60 °C) required to keep the polymerase enzyme active [130]. When using rolling circle amplification (RCA) coupled with nLFA, it made possible the colorimetric detection of synthetic RBD target DNA (16.7 nM) amplified by a biotin primer to a padlock probe specific for RBD (8.33 nM) modified with a FAM probe (0.1 μM), which bound to anti-FAM Ab/AuNPs and was captured by SA on the T-line [131].

## 5. Conclusions and Perspectives

The SARS-CoV-2 virus is a persistent global disease, so prevention and treatment schemes are essential. Even if significant efforts have been made to ensure the rapid detection of viral loads, further improvements are expected, in particular for single-use disposable tests that are accessible to non-medical personnel (e.g., pregnant women [132]). In this review, the author discusses about the advances in the biofunctionalization of gold nanoparticles with different viral proteins against SARS-CoV-2 for the direct screening of positive human samples. At present, only a few studies have used nanomaterials for the biosensing of SARS-CoV-2. In the first part of the review, the author focuses on the applications of AuNPs that have been used for computational predictions against viral infections; meanwhile, in the second part, the author summarizes the use of dry chemistry on solid supports with no rinsing steps and wet “in-tube” chemistry/paper flexible supports with rinsing steps. Finally, AuNPs have been implicated in the development of lateral flow assays [133], producing low-cost tools that are sensitive, specific, user friendly, and less time consuming. In the near future, biofunctionalized AuNPs will be deeply concerned with the development of modern sensing strategies, focusing on the ultrasensitive rapid assays based on SERS-ELISA in plates [134], multiple detection capabilities in LFA-SEM [135], and the simultaneous detection of IgA/IgM/IgG antibodies [136] against SARS-CoV-2, enabling the more effective monitoring of the evolution of the pandemic [137]. Efforts will also focus on the construction of high-throughput quantitative PCR tests for the immediate tracking of SARS-CoV-2 variants [138], as well as the full integration of LFA with biocompatible nanomaterials [139] for better discrimination between false negative results due to low viral loads and false positive results due to a mutant strain in the tested sample. Moreover, the next generation of LFA tests will certainly serve as routine diagnostic tools for vaccine quality control and the monitoring of active antibody titers developed in the vaccinated population, enabling the direct public coordination and understanding of vaccine dose(s). In addition, prevention conditions must be adopted as a permanent priority, with a particular focus on the development of commercial disinfectants based on biocompatible nanopolymers to facilitate smart transport for human activities [140].

In conclusion, remarkable progress has been made during the COVID-19 pandemic, and the world must remain vigilant [141] and prepare for possible future pandemics. To face this uncertainty, nanotechnological [142,143,144] miniaturized portable devices with multidetection [145] and high-throughput multimodal microtiter plate immunoassays [146] will play a central role in enabling the early detection of targeted viral loads that will save precious time during the isolation of patients and the delivery of medical treatment. Moreover, the preparation of AuNPs using green chemistry, which is less toxic to the environment and to humans, may contribute to an increase in the antiviral properties of metal nanoparticles for the future formulation of viral vaccines [147,148].

## Data Availability

Not applicable.

## Abbreviations

AA—amino acids; ACE2—angiotensin-converting enzyme 2; Au-GNPs—polygonal gold nanotags; Au-Ag HNSs—gold–silver alloy hollow nanoshells; AEC—3-amino-9-ethylcarbazole; Av—streptavidin; ASO—unique target regions of the N gene (ASO_1_ starting with 421 and ending with 440; ASO_2_ starting with 443 and ending with 462); DTNB—5,5-dithiobis-2-nitrobenzoic acid; Cys—cysteamine; FAM—6-carboxyfluorescein; GAG—glycosaminoglycans; HEP—heparin; HS—heparin sulfate (HS) proteoglycans; HRP—peroxidase; MBA—4-mercaptobenzoic acid; 4-NBT—4-nitrobenzenethiol; PEG—thiol-PEG-carboxyl; SSC—saline sodium citrate; SA-NC—streptavidin-modified nitrocellulose.
